# Supplementary material for: Stakeholders’ Consensus on Strategies for Self- and Other-Regulation of Video Game Play: A Mixed Methods Study
Source: Int J Environ Res Public Health. 2020 May 28;17(11):3846. doi: 10.3390/ijerph17113846 (PMC7313040; doi:10.3390/ijerph17113846)
Supplement: Supplementary file 1 [file ijerph-17-03846-s001.zip › EV dofile with list.docx]

EV dofile with list

/*******

mcc

make databases

Part 1:

1. Generate variable

2. Enter data directly into Stata data editor for survey vars (only 7 cases!)

3. Save dataset

Part 2. Create datasets for analyzing rankings

2a: Question 1: What features of games make you want to pick up a game or keep playing it?

4. Clear and enter data for 1st question

5. Analyze rankings for first question

6. Save dataset

2b: Question 2: What features of games make you want to stop playing?

7. Clear and enter data for 1st question

8. Analyze rankings for first question

9. Save dataset

2c: Question 3: What are some strategies people can use to regulate their gaming?

10. Clear and enter data for 1st question

11. Analyze rankings for first question

12. Save dataset

2d: Question 4: What features of games make it easier for people to regulate

their gaming--what can game developers add?

13. Clear and enter data for 1st question

14. Analyze rankings for first question

15. Save dataset

V1: 9/2/2017

V2: 12/8/2018 descriptives

***/

/***************************************************************************

PART 1 : CREATE DATASET AND VARS

***************************************************************************/

clear all

cap log close

log using "C:\Users\Michelle\Dropbox\Michelle_Collab_Research\Presentations\Escape_Velocity\Analysis\Logs\analyze_rankingsV2.smcl", replace

set obs 7

generate var1 = 1 in 1

replace var1 = 2 in 2

replace var1 = 13 in 3

replace var1 = 14 in 4

replace var1 = 15 in 5

replace var1 = 26 in 6

replace var1 = 27 in 7

rename var1 ID

cap drop Q1

gen Q1=.

tostring Q1, replace

replace Q1 = "" if Q1 == "."

***Enter data Subject 1

replace Q1 = "1,4,7" if ID==1

rename Q1 group

generate str var3 = "1,4,6,7" if ID==1

generate var4 = 31 if ID==1

generate var5 = 1 if ID==1

generate var6 = 1 if ID==1

generate str var7 = "4" if ID==1

generate var8 = 4 if ID==1

generate var9 = 2 if ID==1

generate var10 = 1 if ID==1

generate var11 = 1 if ID==1

generate var12 = 2.5 if ID==1

generate var13 = 12.5 if ID==1

generate var14 = 3 if ID==1

generate var15 = 300 if ID==1

generate var16 = 3 if ID==1

rename var3 identify

rename var4 age

rename var5 gender

rename var6 sexorien

rename var7 race

rename var8 educ

rename var9 income

rename var10 work

rename var11 playgamY

rename var12 hpd

rename var13 dpw

rename var14 sysowned

rename var15 money

rename var16 playdur

gen Q22=.

foreach var in Q16 Q17 Q18 Q19 Q20 Q21 Q23 Q24 Q25 Q26 Q27 race {

cap drop `var'

gen str2 `var' = ""

}

replace Q16 = "NA" if ID==1

replace Q17 = "Pokemon, Pokemon_GO" if ID==1

replace Q18 = "No_Man's_Sky, The_Witcher_3,Minecraft" if ID==1

replace Q19 = "Half_Life_2, The_Sims,Roller_Coaster_Tycoon" if ID==1

replace Q20 = "Pokemon_series, The_World_Ends_with_You, Dragon_Warrior_Monster" if ID==1

replace Q21 = "No_Man's_Sky, Legend_of_Zelda_Ocarina, Assasin's_Creed_3" if ID==1

replace Q22 = 3 if ID==1

replace Q23 = "Interested in the title and insights into the gaming industry" if ID==1

rename Q16 favcomp

rename Q17 favmobile

rename Q18 favconsole

rename Q19 allfavecomp

rename Q20 allfavemob

rename Q21 allfavecons

rename Q22 gamerID

rename Q23 whycame

rename Q24 Q1rank

rename Q25 Q2rank

rename Q26 Q3rank

rename Q27 Q4rank

codebook Q1rank Q2rank Q3rank Q4rank

replace Q1rank = "3,4,2" if ID==1

replace Q2rank = "6,5,8" if ID==1

replace Q3rank = "9,6,10" if ID==1

replace Q4rank = "11,7,6" if ID==1

replace race= "4" if ID==1

sort ID

***********************Enter data for subject ID 2

replace group = "4,7" if ID==2

replace identify = "4" if ID==2

replace age = 35 if ID==2

replace gender = 1 if ID==2

replace sexorien = 1 if ID==2

replace race = "4" if ID==2

replace educ = 4 if ID==2

replace income = 3 if ID==2

replace work = 1 if ID==2

replace playgamY = 1 if ID==2

replace hpd = 2 if ID==2

replace dpw = 6 if ID==2

replace sysowned = 1 if ID==2

replace money = 100 if ID==2

replace playdur = 2 if ID==2

replace gamerID = 1 if ID==2

replace favcomp = "Rainbow_Six_Seige, Overwatch, Dawn_of_War_III" if ID==2

replace favmobile = "NA" if ID==2

replace favconsole = "NA" if ID==2

replace allfavecomp = "Star_Control_III, Dawn_of_War_II, Indiana_Jones_Atlantis" if ID==2

replace allfavemob = "NA" if ID==2

replace allfavecons = "Final_Fantasy_Tactics, Wipe_Out, Gran_Turismo" if ID==2

replace whycame = "I personally think that I have a healthy attitude towards games, but I'm interested to hear why some people don't, and whether their addiction is specific to games or they have personalities that make them prone to become addiction and it just happened to be games whereas for others it's alcohol, drugs, etc." if ID==2

replace Q1rank = "4,5,1" if ID==2

replace Q2rank = "4,3,8" if ID==2

replace Q3rank = "9,6,10" if ID==2

replace Q4rank = "11,7,6" if ID==2

****************Enter data for subject ID 13

replace group = "4" if ID==13

replace identify = "4" if ID==13

replace age = 27 if ID==13

replace gender = 1 if ID==13

replace sexorien = 3 if ID==13

replace race = "4" if ID==13

replace educ = 3 if ID==13

replace playgamY = 1 if ID==13

replace Q1rank = "2,3,5" if ID==13

replace Q2rank = "6,8,5" if ID==13

replace Q3rank = "9,6,10" if ID==13

replace Q4rank = "11,7,8" if ID==13

*****************Enter data for subject ID 14

*****only answered rankings because came late (didn't submit sticky notes)

replace Q1rank = ".,.,." if ID==14

replace Q2rank = "3,4,." if ID==14

replace Q3rank = "6,10,9" if ID==14

replace Q4rank = "8,11,7" if ID==14

*****************Enter data for subject ID 15

replace group = "1,4,5,7" if ID==15

replace identify = "5" if ID==15

replace age = 30 if ID==15

replace gender = 1 if ID==15

replace sexorien = 1 if ID==15

replace race = "4" if ID==15

replace educ = 6 if ID==15

replace income = 3 if ID==15

replace work = 1 if ID==15

replace playgamY = 2 if ID==15

replace gamerID = 3 if ID==15

replace whycame = "love the topic" if ID==15

replace Q1rank = "5,2,3" if ID==15

replace Q2rank = "6,8,4" if ID==15

replace Q3rank = "9,10,." if ID==15

replace Q4rank = "6,.,." if ID==15

*****************Enter data for subject ID 26

replace group = "1,3,4" if ID==26

replace identify = "3" if ID==26

replace age = 38 if ID==26

replace gender = 2 if ID==26

replace sexorien = 1 if ID==26

replace race = "1,5" if ID==26

replace educ = 6 if ID==26

replace income = 4 if ID==26

replace work = 1 if ID==26

replace playgamY = 1 if ID==26

replace hpd = 5 if ID==26

replace dpw = 4 if ID==26

replace sysowned = 1 if ID==26

replace money = 5000 if ID==26

replace playdur = 2 if ID==26

replace gamerID = 3 if ID==26

replace favcomp = "Jackpot_Slots, War_games" if ID==26

replace favmobile = "Candy_crush, Slots, Pet_Rescue" if ID==26

replace favconsole = "NA" if ID==26

replace allfavecomp = "Slots_of_any_type_design" if ID==26

replace allfavemob = "Candy_crush" if ID==26

replace allfavecons = "NA" if ID==26

replace whycame = "Why increase playing time?" if ID==26

replace Q1rank = "3,5,2" if ID==26

replace Q2rank = "7,3,6" if ID==26

replace Q3rank = "10,6,9" if ID==26

replace Q4rank = "6,11,8" if ID==26

*****************Enter data for subject ID 27

replace group = "4" if ID==27

replace identify = "8" if ID==27

replace age = 49 if ID==27

replace gender = 1 if ID==27

replace sexorien = 1 if ID==27

replace race = "9" if ID==27

replace educ = 6 if ID==27

replace income = 4 if ID==27

replace work = 1 if ID==27

replace playgamY = 1 if ID==27

replace hpd = 3 if ID==27

replace dpw = 6 if ID==27

replace sysowned = 0 if ID==27

replace money = 200 if ID==27

replace playdur = 3 if ID==27

replace gamerID = 1 if ID==27

replace favcomp = "Civilization_series, " if ID==27

replace favcomp = "Civilization_series, Xcom_2, Sim_City_series" if ID==27

replace favmobile = "NA" if ID==27

replace favconsole = "NA" if ID==27

replace allfavecomp = "Sim_City_2000, Civilization_4, Mass_Effect_3" if ID==27

replace allfavemob = "NA" if ID==27

replace allfavecons = "NA" if ID==27

replace whycame = "Curiosity" if ID==27

replace Q1rank = "3,4,5" if ID==27

replace Q2rank = "8,7,3" if ID==27

replace Q4rank = "11,7,6" if ID==27

replace Q3rank = "9,6,." if ID==27

list ID group identify age race educ income hpd gamerID in 1/7

list ID Q1rank Q2rank Q3rank Q4rank

save "C:\Users\Michelle\Dropbox\Michelle_Collab_Research\Presentations\Escape_Velocity\Analysis\Data\EV_surveysV2.dta", replace

/***************************************************************************

PART 2. Create datasets for analyzing Q1-Q4

************************************************************************/

/*

ID gender work sysowned favcomp allfavemob Q2rank

group sexorien playgamY money favmobile allfavecons Q3rank

identify educ hpd playdur favconsole whycame Q4rank

age income dpw gamerID allfavecomp Q1rank race

*/

***create separate vars for each "choose all"

foreach var in group identify race favcomp Q1rank allfavemob Q2rank ///

favmobile allfavecons Q3rank favconsole Q4rank allfavecomp {

split `var', parse(,)

}

codebook, compact

ds group1- allfavecomp3

ds group1- allfavecomp3

/* group1 identify3 favcomp3 allfavemob3 favmobile3 Q3rank3 Q4rank3

group2 identify4 Q1rank1 Q2rank1 allfavecons1 favconsole1 allfavecomp1

group3 race1 Q1rank2 Q2rank2 allfavecons2 favconsole2 allfavecomp2

group4 race2 Q1rank3 Q2rank3 allfavecons3 favconsole3 allfavecomp3

identify1 favcomp1 allfavemob1 favmobile1 Q3rank1 Q4rank1

identify2 favcomp2 allfavemob2 favmobile2 Q3rank2 Q4rank2 */

foreach var in group1 group2 group3 group4 identify1 identify2 identify3 identify4 ///

race1 race2 Q1rank1 Q1rank2 Q1rank3 Q2rank1 Q2rank2 Q2rank3 ///

Q3rank1 Q3rank2 Q3rank3 Q4rank1 Q4rank2 Q4rank3 {

destring `var', gen (`var'num)

tab `var' `var'num

}

codebook *num

lab def group 1 "1.res" 3 "3.edu" 4 "4.game" 5 "5.indus" 6 "6.pol" 7 "7.fam/fr" 8 "8.none" 9 "9.ref", replace

foreach var in group1num group2num group3num group4num identify1num identify2num identify3num identify4num {

lab values `var' group

}

codebook group1num group2num group3num group4num identify1num identify2num identify3num identify4num

cap drop resgrp

egen resgrp=anycount(group1num group2num group3num group4num), values(1)

cap drop edugrp

egen edugrp=anycount(group1num group2num group3num group4num), values(3)

cap drop gamegrp

egen gamegrp=anycount(group1num group2num group3num group4num), values(4)

cap drop indusgrp

egen indusgrp=anycount(group1num group2num group3num group4num), values(5)

cap drop pol

egen pol=anycount(group1num group2num group3num group4num), values(6)

cap drop famgrpr

egen famgrpr=anycount(group1num group2num group3num group4num), values(7)

cap drop nonegrp

egen nonegrp=anycount(group1num group2num group3num group4num), values(8)

cap drop refgrp

egen refgrp=anycount(group1num group2num group3num group4num), values(9)

cap drop resid

egen resid=anycount(identify1num identify2num identify3num identify4num), values(1)

cap drop eduid

egen eduid=anycount(identify1num identify2num identify3num identify4num), values(3)

cap drop gameid

egen gameid=anycount(identify1num identify2num identify3num identify4num), values(4)

cap drop indusid

egen indusid=anycount(identify1num identify2num identify3num identify4num), values(5)

cap drop polid

egen polid=anycount(identify1num identify2num identify3num identify4num), values(6)

cap drop famid

egen famid=anycount(identify1num identify2num identify3num identify4num), values(7)

cap drop noneid

egen noneid=anycount(identify1num identify2num identify3num identify4num), values(8)

cap drop refid

egen refid=anycount(identify1num identify2num identify3num identify4num), values(9)

codebook group*num ident*num

cap drop white

egen white=anycount(race1num race2num), values(4)

cap drop AmInd

egen AmInd=anycount(race1num race2num), values (1)

cap drop black

egen black=anycount(race1num race2num), values(5)

codebook white black AmInd

lab def theme 1 "1.compet" 2 "2.game" 3 "3.immers" 4 "4.mood" 5 "5.ach" 6 "6.soc" ///

7 "7.env" 8 "8.novel" 9 "9.selfreg" 10 "10.analyz" 11 "11.mech", replace

foreach var in Q1rank1num Q1rank2num Q1rank3num Q2rank1num Q2rank2num Q2rank3num ///

Q3rank1num Q3rank2num Q3rank3num Q4rank1num Q4rank2num Q4rank3num {

lab values `var' theme

}

codebook Q*num

save "C:\Users\Michelle\Dropbox\Michelle_Collab_Research\Presentations\Escape_Velocity\Analysis\Data\EV_surveys_expV2.dta", replace

/***************************************************************************

PART 2a. Create dataset for analyzing Q1

***************************************************************************/

keep ID Q1*num

list

save "C:\Users\Michelle\Dropbox\Michelle_Collab_Research\Presentations\Escape_Velocity\Analysis\Data\EV_Q1V2.dta", replace

*use "C:\Users\Michelle\Dropbox\Michelle_Collab_Research\Presentations\Escape_Velocity\Analysis\Data\EV_Q1.dta", clear

list

list, nolab

****generate the list of themes that is the domain for the question

cap drop rcimmers

gen rcimmers=.

cap drop rcmood

gen rcmood=.

cap drop rcgame

gen rcgame=.

cap drop rcachiev

gen rcachiev=.

cap drop rccompet

gen rccompet=.

list

list, nolab

***replace theme variables with their rankings for each person

replace rcimmers=1 if Q1rank1num==3

replace rcimmers=2 if Q1rank2num==3

replace rcimmers=3 if Q1rank3num==3

list

list, nolab

replace rcmood=1 if Q1rank1num==4

replace rcmood=2 if Q1rank2num==4

replace rcmood=3 if Q1rank3num==4

replace rcgame=1 if Q1rank1num==2

replace rcgame=2 if Q1rank2num==2

replace rcgame=3 if Q1rank3num==2

replace rcachiev=1 if Q1rank1num==5

replace rcachiev=2 if Q1rank2num==5

replace rcachiev=3 if Q1rank3num==5

replace rccompet=1 if Q1rank1num==1

replace rccompet=2 if Q1rank2num==1

replace rccompet=3 if Q1rank3num==1

list

list, nolab

****debugging--dropping Q1 before reshape

drop Q1*

*****GET THE # OBS FOR TABLE FOR PRESENTATION

codebook, compact

*****I feel like that was the hard way to do it...

reshape long rc, i(ID) j(criterion) string

list

list, nolab

***debugging--rev code before changing missing to 0

cap drop rev

gen rev=4-rc

codebook rev

tab rev rc, mi

recode rev .=0 // not in top 10 means bottom Ö cannot assume missing at random

*at this point you could do a friedman test because there are no longer any ///

* missing data, so no need to use skilmack, but if you wanted to ///

*(it is equivalent to friedman test, and the user written friedman test requires ///

*data to be in a weird format from memory), here is what you need to do

encode criterion, gen(criterion_num) // because skilmack command probably needs ///

* to work with numeric rather than string treatment

set matsize 11000

skilmack rev, id(ID) repeated(criterion_num)

*I guess your focus would more be on Weighted Sum of Centered Ranks than p-value

tab rev criterion

*a very nice way to present your data Iíd have thought

save "C:\Users\Michelle\Dropbox\Michelle_Collab_Research\Presentations\Escape_Velocity\Analysis\Data\EV_Q1V2.dta", replace

use "C:\Users\Michelle\Dropbox\Michelle_Collab_Research\Presentations\Escape_Velocity\Analysis\Data\EV_surveys_expV2.dta", clear

/***************************************************************************

PART 2b. Create dataset for analyzing Q2

***************************************************************************/

keep ID Q2*num

list

save "C:\Users\Michelle\Dropbox\Michelle_Collab_Research\Presentations\Escape_Velocity\Analysis\Data\EV_Q2V2.dta", replace

list

list, nolab

****generate the list of themes that is the domain for the question:

cap drop rcimmers

gen rcimmers=.

cap drop rcmood

gen rcmood=.

cap drop rcsoc

gen rcsoc=.

cap drop rcachiev

gen rcachiev=.

cap drop rcnovel

gen rcnovel=.

cap drop rcenv

gen rcenv=.

list

list, nolab

codebook, compact

***replace theme variables with their rankings for each person

replace rcimmers=1 if Q2rank1num==3

replace rcimmers=2 if Q2rank2num==3

replace rcimmers=3 if Q2rank3num==3

list

list, nolab

replace rcmood=1 if Q2rank1num==4

replace rcmood=2 if Q2rank2num==4

replace rcmood=3 if Q2rank3num==4

replace rcsoc=1 if Q2rank1num==6

replace rcsoc=2 if Q2rank2num==6

replace rcsoc=3 if Q2rank3num==6

replace rcachiev=1 if Q2rank1num==5

replace rcachiev=2 if Q2rank2num==5

replace rcachiev=3 if Q2rank3num==5

replace rcnovel=1 if Q2rank1num==8

replace rcnovel=2 if Q2rank2num==8

replace rcnovel=3 if Q2rank3num==8

replace rcenv=1 if Q2rank1num==7

replace rcenv=2 if Q2rank2num==7

replace rcenv=3 if Q2rank3num==7

list

list, nolab

****debugging--dropping Q2 before reshape

drop Q2*

*****GET THE # OBS FOR TABLE FOR PRESENTATION

codebook, compact

codebook, compact

*****GET THE # OBS FOR TABLE FOR PRESENTATION

*****I feel like that was the hard way to do it...

reshape long rc, i(ID) j(criterion) string

list

list, nolab

***debugging--rev code before changing missing to 0

cap drop rev

gen rev=4-rc

codebook rev

tab rev rc, mi

recode rev .=0 // not in top 10 means bottom Ö cannot assume missing at random

*at this point you could do a friedman test because there are no longer any ///

* missing data, so no need to use skilmack, but if you wanted to ///

*(it is equivalent to friedman test, and the user written friedman test requires ///

*data to be in a weird format from memory), here is what you need to do

encode criterion, gen(criterion_num) // because skilmack command probably needs ///

* to work with numeric rather than string treatment

set matsize 11000

skilmack rev, id(ID) repeated(criterion_num)

*I guess your focus would more be on Weighted Sum of Centered Ranks than p-value

******GET THE SKILMACK WSCR FOR TABLE

tab rev criterion

*a very nice way to present your data Iíd have thought

save "C:\Users\Michelle\Dropbox\Michelle_Collab_Research\Presentations\Escape_Velocity\Analysis\Data\EV_Q2V2.dta", replace

use "C:\Users\Michelle\Dropbox\Michelle_Collab_Research\Presentations\Escape_Velocity\Analysis\Data\EV_surveys_expV2.dta", clear

/***************************************************************************

PART 2c. Create dataset for analyzing Q3

***************************************************************************/

keep ID Q3*num

list

save "C:\Users\Michelle\Dropbox\Michelle_Collab_Research\Presentations\Escape_Velocity\Analysis\Data\EV_Q3V2.dta", replace

use "C:\Users\Michelle\Dropbox\Michelle_Collab_Research\Presentations\Escape_Velocity\Analysis\Data\EV_Q3.dta", clear

****generate the list of themes that is the domain for the question:

cap drop rcsoc

gen rcsoc=.

cap drop rcselfreg

gen rcselfreg=.

cap drop rcanalyze

gen rcanalyze=.

list

list, nolab

codebook, compact

***replace theme variables with their rankings for each person

replace rcsoc=1 if Q3rank1num==6

replace rcsoc=2 if Q3rank2num==6

replace rcsoc=3 if Q3rank3num==6

replace rcselfreg=1 if Q3rank1num==9

replace rcselfreg=2 if Q3rank2num==9

replace rcselfreg=3 if Q3rank3num==9

replace rcanalyze=1 if Q3rank1num==10

replace rcanalyze=2 if Q3rank2num==10

replace rcanalyze=3 if Q3rank3num==10

list

list, nolab

****debugging--dropping Q3 before reshape

drop Q3*

*****GET THE # OBS FOR TABLE FOR PRESENTATION

codebook, compact

*****GET THE # OBS FOR TABLE FOR PRESENTATION

*****I feel like that was the hard way to do it...

reshape long rc, i(ID) j(criterion) string

list

list, nolab

***debugging--rev code before changing missing to 0

cap drop rev

gen rev=4-rc

codebook rev

tab rev rc, mi

recode rev .=0

tab rev rc, mi

// not in top 10 means bottom Ö cannot assume missing at random

*at this point you could do a friedman test because there are no longer any ///

* missing data, so no need to use skilmack, but if you wanted to ///

*(it is equivalent to friedman test, and the user written friedman test requires ///

*data to be in a weird format from memory), here is what you need to do

encode criterion, gen(criterion_num) // because skilmack command probably needs ///

* to work with numeric rather than string treatment

set matsize 11000

skilmack rev, id(ID) repeated(criterion_num)

*I guess your focus would more be on Weighted Sum of Centered Ranks than p-value

******GET THE SKILMACK WSCR FOR TABLE

tab rev criterion

*a very nice way to present your data Iíd have thought

save "C:\Users\Michelle\Dropbox\Michelle_Collab_Research\Presentations\Escape_Velocity\Analysis\Data\EV_Q3V2.dta", replace

use "C:\Users\Michelle\Dropbox\Michelle_Collab_Research\Presentations\Escape_Velocity\Analysis\Data\EV_surveys_exp.dta", clear

/***************************************************************************

PART 2d. Create dataset for analyzing Q4

***************************************************************************/

keep ID Q4*num

list

save "C:\Users\Michelle\Dropbox\Michelle_Collab_Research\Presentations\Escape_Velocity\Analysis\Data\EV_Q4V2.dta", replace

use "C:\Users\Michelle\Dropbox\Michelle_Collab_Research\Presentations\Escape_Velocity\Analysis\Data\EV_Q4.dta", clear

****generate the list of themes that is the domain for the question:

cap drop rcsoc

gen rcsoc=.

cap drop rcnovel

gen rcnovel=.

cap drop rcenv

gen rcenv=.

cap drop rcmech

gen rcmech=.

list

list, nolab

codebook, compact

***replace theme variables with their rankings for each person

replace rcmech=1 if Q4rank1num==11

replace rcmech=2 if Q4rank2num==11

replace rcmech=3 if Q4rank3num==11

replace rcsoc=1 if Q4rank1num==6

replace rcsoc=2 if Q4rank2num==6

replace rcsoc=3 if Q4rank3num==6

replace rcnovel=1 if Q4rank1num==8

replace rcnovel=2 if Q4rank2num==8

replace rcnovel=3 if Q4rank3num==8

replace rcenv=1 if Q4rank1num==7

replace rcenv=2 if Q4rank2num==7

replace rcenv=3 if Q4rank3num==7

list

list, nolab

****debugging--dropping Q4 before reshape

drop Q4*

*****GET THE # OBS FOR TABLE FOR PRESENTATION

codebook, compact

*****I feel like that was the hard way to do it...

reshape long rc, i(ID) j(criterion) string

list

list, nolab

***debugging--rev code before changing missing to 0

cap drop rev

gen rev=4-rc

codebook rev

tab rev rc, mi

recode rev .=0

tab rev rc, mi

// not in top 10 means bottom Ö cannot assume missing at random

*at this point you could do a friedman test because there are no longer any ///

* missing data, so no need to use skilmack, but if you wanted to ///

*(it is equivalent to friedman test, and the user written friedman test requires ///

*data to be in a weird format from memory), here is what you need to do

encode criterion, gen(criterion_num) // because skilmack command probably needs ///

* to work with numeric rather than string treatment

set matsize 11000

skilmack rev, id(ID) repeated(criterion_num)

*I guess your focus would more be on Weighted Sum of Centered Ranks than p-value

******GET THE SKILMACK WSCR FOR TABLE

tab rev criterion

*a very nice way to present your data Iíd have thought

save "C:\Users\Michelle\Dropbox\Michelle_Collab_Research\Presentations\Escape_Velocity\Analysis\Data\EV_Q4V2.dta", replace

use "C:\Users\Michelle\Dropbox\Michelle_Collab_Research\Presentations\Escape_Velocity\Analysis\Data\EV_surveys_exp.dta", clear

codebook, compact
